# Meta-analysis of clinical data using human meiotic genes identifies a novel cohort of highly restricted cancer-specific marker genes

**DOI:** 10.18632/oncotarget.580

**Published:** 2012-08-13

**Authors:** Julia Feichtinger, Ibrahim Aldeailej, Rebecca Anderson, Mikhlid Almutairi, Ahmed Almatrafi, Naif Alsiwiehri, Keith Griffiths, Nicholas Stuart, Jane A. Wakeman, Lee Larcombe, Ramsay J. McFarlane

**Affiliations:** ^1^ North West Cancer Research Fund Institute, Bangor University, Bangor, LL57 2UW, UK; ^2^ Therapies and Health Sciences, Betsi Cadwaladr University Health Board, Bangor, LL57 2UW, UK; ^3^ Medical Sciences, Bangor University, Bangor, LL57 2UW, UK; ^4^ Bioinformatics Group, Cranfield Health, Cranfield University, Beds, MK43 0AL, UK; ^5^ NISCHR Cancer Genetics Biomedical Research Unit

**Keywords:** cancer biomarkers, cancer testes antigens, oncogenes, meiosis, PRDM9, cohesins drug targets

## Abstract

Identifying cancer-specific biomarkers represents an ongoing challenge to the development of novel cancer diagnostic, prognostic and therapeutic strategies. Cancer/testis (CT) genes are an important gene family with expression tightly restricted to the testis in normal individuals but which can also be activated in cancers. Here we develop a pipeline to identify new CT genes. We analysed and validated expression profiles of human meiotic genes in normal and cancerous tissue followed by meta-analyses of clinical data sets from a range of tumour types resulting in the identification of a large cohort of highly specific cancer biomarker genes, including the recombination hot spot activator *PRDM9* and the meiotic cohesin genes *SMC1beta* and *RAD21L*. These genes not only provide excellent cancer biomarkers for diagnostics and prognostics, but may serve as oncogenes and have excellent drug targeting potential.

## INTRODUCTION

The demarcation of neoplastic cells from healthy tissue represents an important goal in clinical oncology; this is of particular interest given the need for diagnostic markers to enable early intervention strategies, such as surgical resection, and the re-emergence of immunotherapeutics, cancer vaccines and targeted drug delivery via antibody-drug conjugates [1-10]. To achieve this goal, the identification of tumour-associated antigens is of central importance [for example, see 11-13]. Whilst almost all cancer cells have an altered gene expression profile, including many up regulated genes, most of the associated antigens are recognised as ‘self’ by the immune system, limiting their use in immune therapeutic, prognostic and diagnostic technologies. One family of proteins, the so-called cancer/testis (CT) antigens, represents an excellent group of cancer-specific biomarkers [14-21]. These are produced in the testes of healthy male adults and can also be found in cells with a cancerous phenotype. The immunological privilege of the testis [22,23] makes the CT antigens excellent immunological targets and a number of CT antigens have been employed successfully in a range of clinical applications, including adoptive therapeutics for late stage cancer treatment [for example, see 24]. Some CT antigens are also present in other immunologically privileged tissues of the central nervous system (CNS) and these are referred to as cancer/testis-CNS (CT/CNS) antigens [25].

Many genes have been purported to encode CT antigens [21], however, not all of these have endured continued scrutiny and many of the genes have subsequently been found to have some degree of expression in normal somatic tissues [25]. This has lead to the redefining of CT genes into testis (and CNS)-restricted and testis (and CNS)-selective, where there is some evidence to indicate the latter class are expressed in at least one non-immune privileged, normal tissue type [25,26].

CT antigens have been further sub-classified into those which are encoded by genes on the X chromosome (X-CT genes) and those which are encoded by genes on autosomes (non-X-CT genes) [14-21]. The majority of characterised testis-restricted CT genes are X-CT genes and many of these reside within large families of orthologous genes, such as the *MAGE* family [14-21,27]. In addition, some CT genes are co-expressed in the same cancerous tissue, suggesting a dysfunction in one or more, as yet uncharacterised, testis-specific transcriptional regulatory pathway(s) [for example, see 28].

It has been demonstrated that some CT antigens have the potential for oncogenic activity or contribute to maintaining or enhancing the neoplastic state [19]. For example, MAGE-A2 has been demonstrated to induce the down regulation of one of the primary tumour suppressor genes, *p53* [29]. Furthermore, MAGE-A2 and another MAGE family member, MAGE-A6, have been demonstrated to have the potential to induce resistance to chemotherapeutic agents [30]. However, the function, the oncogenic activity and the drug resistance-inducing potential of CT antigens remains poorly studied considering the potential importance of these proteins.

There has been speculation that some CT antigens could function in the testes to mediate the meiotic programme [31,32]. During meiosis the chromosomes of diploid progenitor cells (spermatogonia in testis) become reductionally segregated to produce haploid gametes (sperm cells in testis) [33,34]. This meiotic chromosome segregation involves a complex and poorly understood series of events, which include the pairing of homologous chromosomes followed by a covalent conjoining to generate a bivalent which is required for chromatid alignment at the first meiotic division. It has been postulated that the aberrant production of CT antigens with chromosome modulating potential in mitotically dividing somatic cells could result in inappropriate non-allelic inter-/intra-chromosomal recombination and inter-homologue recombination events which could generate oncogenic genetic changes such as translocations and losses of heterozygocity [21,31,32]. In addition, the aberrant expression of meiotic chromosome regulators in matched induced pluripotent stem cells (iPSCs) has been demonstrated to illicit an immune response to iPSC-induced teratomas in mice indicating a broader importance to understanding the consequences of aberrant expression of meiotic genes [35].

In male mammals there exists a unique mechanism for the meiosis-specific transcriptional silencing of the X chromosome during the meiotic zygotene to pachytene transition, which is dependent upon meiotic double-strand break formation in unpaired chromatin [36]. This meiotic X inactivation suggests that most of the genes encoding known testis-restricted CT antigens are silenced during meiosis, as most of these are X-encoded and so may have largely non-meiotic roles in the testis.

These findings lead us to postulate that there is a family of human meiosis-specific genes, which are autosomally encoded and therefore not subjected to meiotic X inactivation. If these genes are aberrantly expressed in cancers and iPSCs they might represent a clinically important, novel sub-class of the testis-restricted CT gene family. Moreover, we speculated that such genes might have oncogenic activity by encoding proteins which interfere with chromosome dynamics and cell division when aberrantly expressed in mitotically dividing somatic cells. Here we identify human meiosis-specific genes showing the characteristics of CT genes, which we designate meiCT genes. This work defines a novel, meiosis-specific sub-class of clinically-relevant CT genes which includes previously uncharacterised human testis-specific genes, the human meiotic hotspot regulator gene *PRDM9*, the meiotic regulator gene *STRA8* and meiosis-specific sister chromatid cohesion regulator genes.

## RESULTS

### Analysis of selected meiotic chromosome regulatory genes for CT gene candidature

**Figure 1:**
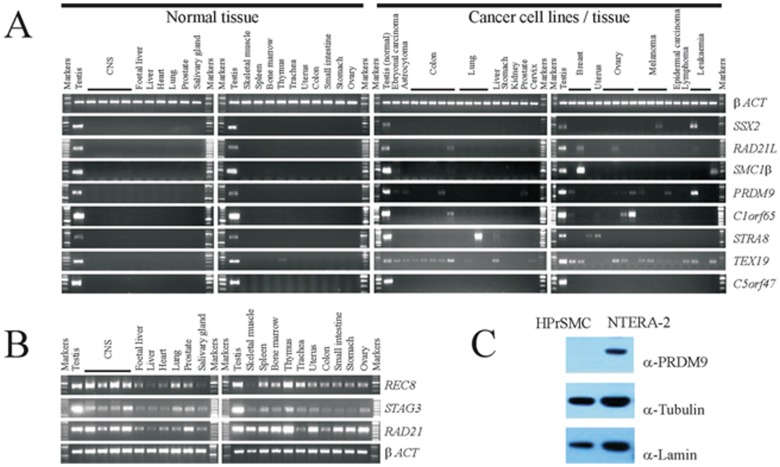
Examples of gene expression and protein production profiles for predicted meiCT genes A. Agarose gels showing RT-PCR profiles generated from a range of normal human tissues obtained *post mortem* (left two panels) and cDNA generated from a range of cancer cell lines or solid tumours (right hand two panels). The expression profile for *betaACT* is a positive control (top row). The profile for *SSX2* provides an example of a previously characterised X-CT gene. Five examples of testis-restricted meiCT genes are shown (*RAD21L/SMC1beta/PRDM9/C1orf65/STRA8*) along with the expression profile of one testis selective meiCT gene, *TEX19*. The *C5orf47* profile provides an example of genes which were testis-restricted with no evidence of expression in any of the cancer cells tested. B. Agarose gels showing RT-PCR profiles for normal human tissues for the mitotic cohesion gene *RAD21* and the two cohesin genes *REC8* and *STAG3*. C. Western blots showing the presence of the PRDM9 protein in the cancer cell line NTERA-2, but not in primary cultures of human prostate smooth muscle cells (HPrSMC).

Some important meiosis-specific genes which encode chromosome modulators have previously been reported to be CT genes, including *SPO11* which encodes a meiosis-specific nuclease required for the initiation of meiotic recombination [15]; however, many of these previously identified meiotic regulators (including, *SPO11, HORMAD1, SYCE1, SYCP1*) have subsequently been found to be selective in their expression profile, suggesting they are not strictly testis-restricted [25]. As a first step to address the possibility that additional meiosis-specific genes might encode highly restricted CT antigens, we selected from the literature a sample cohort of human genes predicted to have meiosis-specific expression (Supplementary Table S1). These included genes encoding subunits of the meiosis-specific cohesin complex (*REC8, STAG3, SMC1beta, RAD21L*), which is responsible for modulating meiotic recombination, meiotic centromere monopolarity and meiotic sister chromatid cohesion [37,38]. To assess the meiotic specificity of the selected genes, we obtained RNA extracted from a panel of normal human tissues, including testis, ovary and CNS tissue. We designed intron-spanning primer sets for the genes of interest and carried out reverse transcriptase polymerase chain reaction (RT-PCR) analysis as described in the materials and methods. Surprisingly, a number of these genes were expressed in a wide range of normal tissues, suggesting that their expression is not exclusively meiosis-specific (Fig. 1B; Supplementary Fig. S1A). These included two genes encoding cohesin components, *REC8* and *STAG3*, the protein products of which are widely accepted as being meiosis-specific from studies in other organisms, including the mouse [for example, 39]. Taking this further, we detected some expression of both *REC8* and *STAG3* in mouse non-meiotic somatic tissue using our RT-PCR conditions (Supplementary Fig. S1B), suggesting that expression of these genes is not fully restricted to meiosis, rather, they are subjected to a meiotic up regulation, consistent with previous analyses in the mouse [for example, 40]. DNA sequencing was used to confirm the identity of all RT-PCR products from both mouse and human. RT-PCR followed by DNA sequencing confirmed that a number of other genes we had postulated would be testis / meiosis-specific were expressed more extensively in non-meiotic tissues (Supplementary Fig. S1A; Supplementary Table S1). Despite the detection of *REC8* and *STAG3* expression in normal tissue, the other two meiosis cohesin genes tested, *RAD21L* and *SMC1beta*, exhibited a tight testis-specific expression profile (Fig. 1A). To explore whether these testis-specific cohesin genes could potentially encode CT antigens we analysed their expression using RT-PCR on RNA samples taken from cancer cell lines and tumour samples from a range of cancer types. For both genes, expression was detected in a number of the cancer cells indicating they are CT genes (Fig. 1A). Of the other manually selected genes a further five exhibited a CT gene expression profile (a mixture of cancer/testis-restricted and cancer/testis/CNS-restricted); these were the meiotic recombination hotspot activator gene *PRDM9*, the nuclear protein in testis (*NUT*) gene, the testis-specific serine/threonine kinase 1 (*TSSK1*) gene, the synaptonemal complex component gene *SYCP1* (which has previously been reported as a CT gene [18]) and the meiotic regulatory gene *STRA8* (Fig. 1A). A sixth gene, *TEX19*, exhibited a testis-selective expression profile as it was expressed in the testis and the thymus, the latter being a tissue known to undergo atrophy in older individuals such as those from which this tissue was derived, which may account for the expression of this gene in the thymus; *TEX19* also exhibited extensive expression in many cancer types (Fig. 1A).

To address whether an expressed gene might be translated into a protein product, which might therefore provide an antigenic target in clinical applications, we carried out western blot analysis to detect the protein product of one of the CT genes identified above, *PRDM9*, for which commercial antibodies were available. The intracellular nature of these antigens does not preclude them from serving as targets for monoclonal antibody therapies or other immunotherapeutic approaches [for example, 24,41,42]. We generated whole cell extracts (WCEs) from one of the cancer cell lines in which *PRDM9* gene expression had been observed, NTERA-2 (Fig. 1A), and a culture of non-cancerous primary human prostate smooth muscle (HPrSM) cells, in which no *PRDM9* gene expression could be detected by RT-PCR. PRDM9 was readily detectable in the cancer cells, but not in the HPrSM cells (Fig. 1C) demonstrating that the expression of the *PRDM9* CT gene results in protein production, leading to the possibility that the de-repression of the *PRDM9* gene generates a protein which could be antigenic and thus be of clinical and oncogenic importance.

### Identification and validation of novel meiosis-associated CT genes using computational analysis of EST data

**Figure 2:**
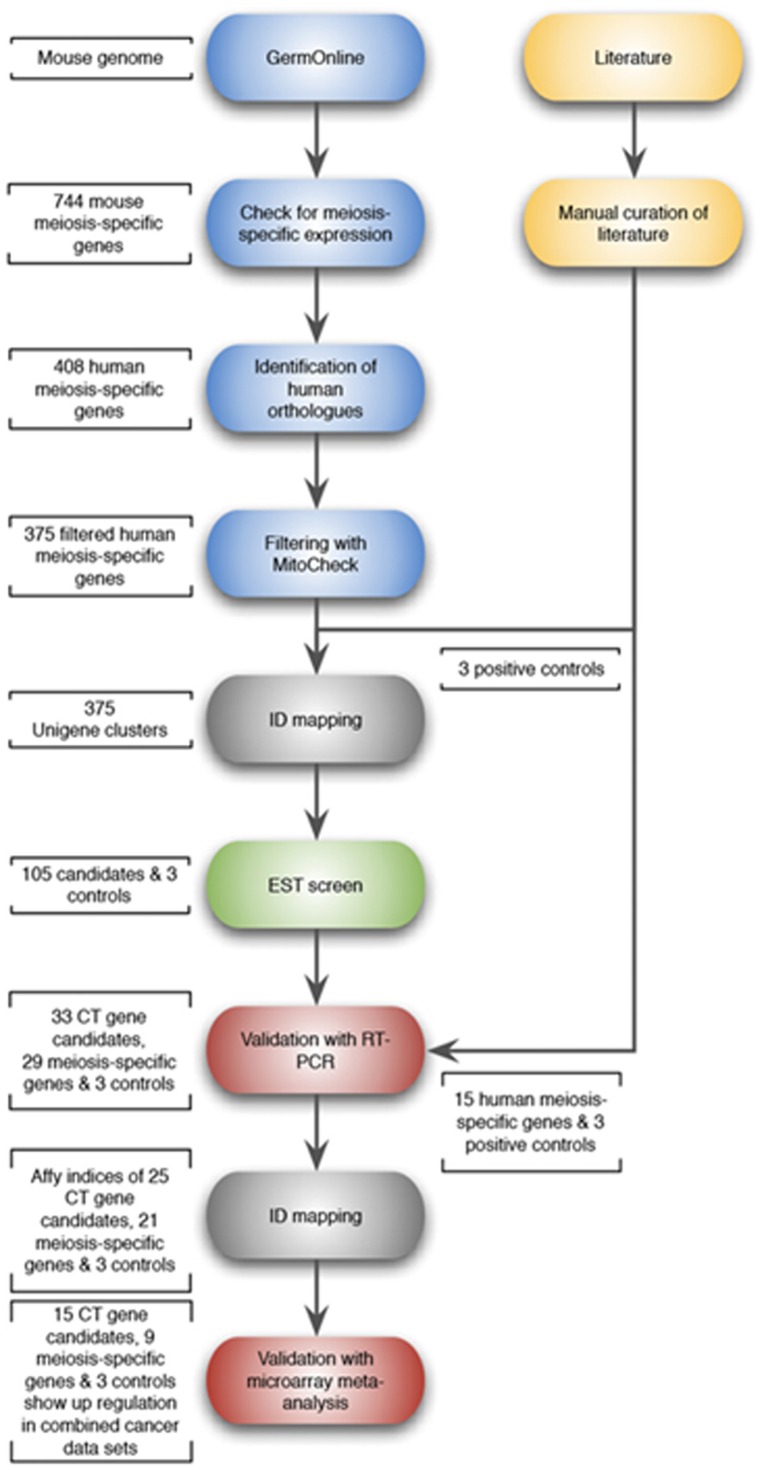
Schematic flow diagram of the approach used for the selection of candidate meiCT genes. Based on a large scale microarray study [43], 744 mouse meiosis-specific genes were selected as a starting point: 408 human orthologues could be identified and 375 human meiosis-specific genes remained after filtering to eliminate non-testis specific genes. All 375 candidates as well as 3 controls (*MAGE-A1, GAGE1* and *SSX2*) were fed into the EST analysis pipeline, which returned 105 candidate genes which were subjected to RT-PCR validation/microarray meta-analysis. Support text to Fig. 2 is given in Supplementary Information.

Whilst the above approach has identified a number of new CT genes, it is limited by the fact that a manual curation of the literature is not only time-consuming, but also exposes relatively few mammalian meiosis-specific genes. We took advantage of a previous large scale microarray study which identified an extensive cohort of genes with expression associated specifically with meiosis and spermatocyte development in mammals [43]. We used this to conduct a systematic approach to identify new meiosis-associated human CT genes. The mouse study provided a starting point of 744 mammalian meiosis-specific genes. After human orthologue assignment and filtering to eliminate non-testis-specific genes, 375 human genes remained and these were fed into an expressed sequence tag (EST) analysis pipeline based on the complete Unigene database (Fig. 2; support text to Fig. 2 is given in Supplementary Information). Briefly, if a candidate gene is represented in a non-testis / non-CNS normal tissue EST library, it was dismissed. The remaining genes were further assessed for representation in cancer EST libraries. This screen identified 177 candidate genes, of which 9 were cancer/testis-restricted (class 1), 75 were testis-restricted (class 2), 21 were cancer/testis/CNS-restricted (class 3) and 72 were testis/CNS-restricted (class 4). We favoured an EST screen, since microarray technology is limited by the number of published cancer arrays available as well as by the number of genes which can be analysed due to lack of gene coverage on arrays. Moreover, an EST screen can confirm the testis-restricted expression pattern and therefore functions as an additional filter to eliminate non-testis-restricted genes. However, microarray data sets were not ignored and following experimental validation of the candidates (see below) we carried out meta-analysis of clinically-relevant cancer microarray data sets (see below).

**Figure 3:**
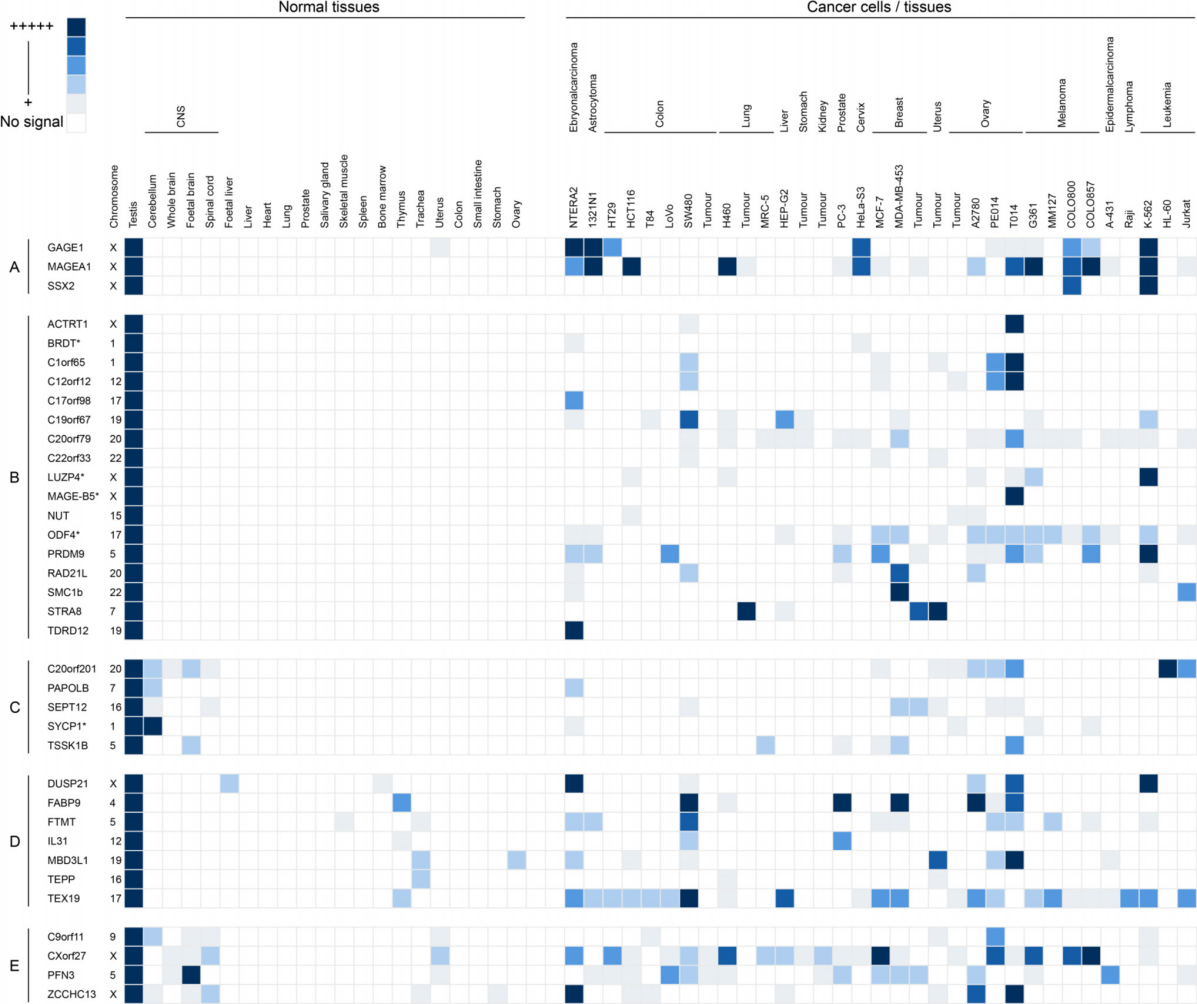
Grid representation of gene expression profiles for the 33 meiCT genes identified. Each gene has a lane allocation on the grid; the presence of a blue square within any column in a given lane represents the presence of an RT-PCR product indicating gene expression. The shade of blue is a qualitative representation of the RT-PCR product intensity on agarose gels. The meiCT genes have been separated into distinct classes based on those of Hofmann *et al.* [25]: A. Examples of known X-CT genes (positive controls); B. Testis-restricted meiCT genes (17 genes); C. Testis/CNS-restricted meiCT genes (5 genes); D. Testis-selective meiCT genes (7 genes); E. Testis/CNS-selective meiCT genes (4 genes). The chromosomal location of all genes is given following the gene name. Genes marked with an asterisk are genes we identified which have previously reported as CT genes [15].

Having identified candidates in the four classes outlined above we validated those in classes 1-3 using RT-PCR. We included the genes in class 2, which are predicted to be testis-specific, but have not been identified in the EST data sets of cancer tissue. We initially carried out RT-PCR on RNA isolated from a range of normal human tissues, including testis-derived RNA, as described above. Of the 105 genes in classes 1-3 we could not obtain RT-PCR products in our control tissue (testis) for 12 genes, resulting in 93 genes which were subjected to RT-PCR validation. Of these, 39 genes were expressed in more than two non-testis/CNS normal tissues and were therefore dismissed at this stage. Of the remaining 54 genes, 41 had expression restricted to testis in normal tissues, 3 had expression restricted to testis and CNS tissue and 10 were testis-specific, or testis/CNS-specific and yet exhibited expression in one or two normal tissues. RT-PCR analysis was carried out to assess the expression profiles of these 54 genes in cancer cells, as above. From these analyses it was determined that 29 genes exhibited no expression in any of the cancerous material and appeared to be tightly testis-specific (Supplementary Table S2; the example of *C5orf47* is shown in Fig. 1A); 12 were CT-restricted genes and they were expressed in the testis and at least one cancer type (the example of *C1orf65* is shown in Fig. 1A; Fig. 3); 3 were cancer/testis/CNS-restricted genes as they were expressed in testis, CNS and at least one cancer type (Fig. 3); 6 were cancer/testis-selective as they were expressed in testis, one or two other non-testis/CNS normal tissues and at least one cancer type (Fig. 3); 4 were cancer/testis/CNS-selective as they were expressed in testis, CNS tissue, one or two other normal tissue types and at least one cancer type (Fig. 3). This resulted in the identification of a total of 25 genes distributed in the various CT classes. This, in combination with the 8 CT genes identified in the preliminary study (see above) resulted in the identification of 33 restricted / selective CT or CT/CNS genes, most of which have not been previously characterised as CT genes and are largely autosomally encoded; we will refer to these as meiCT genes (Fig. 3).

### Meta-analysis of validated candidate genes

**Figure 4:**
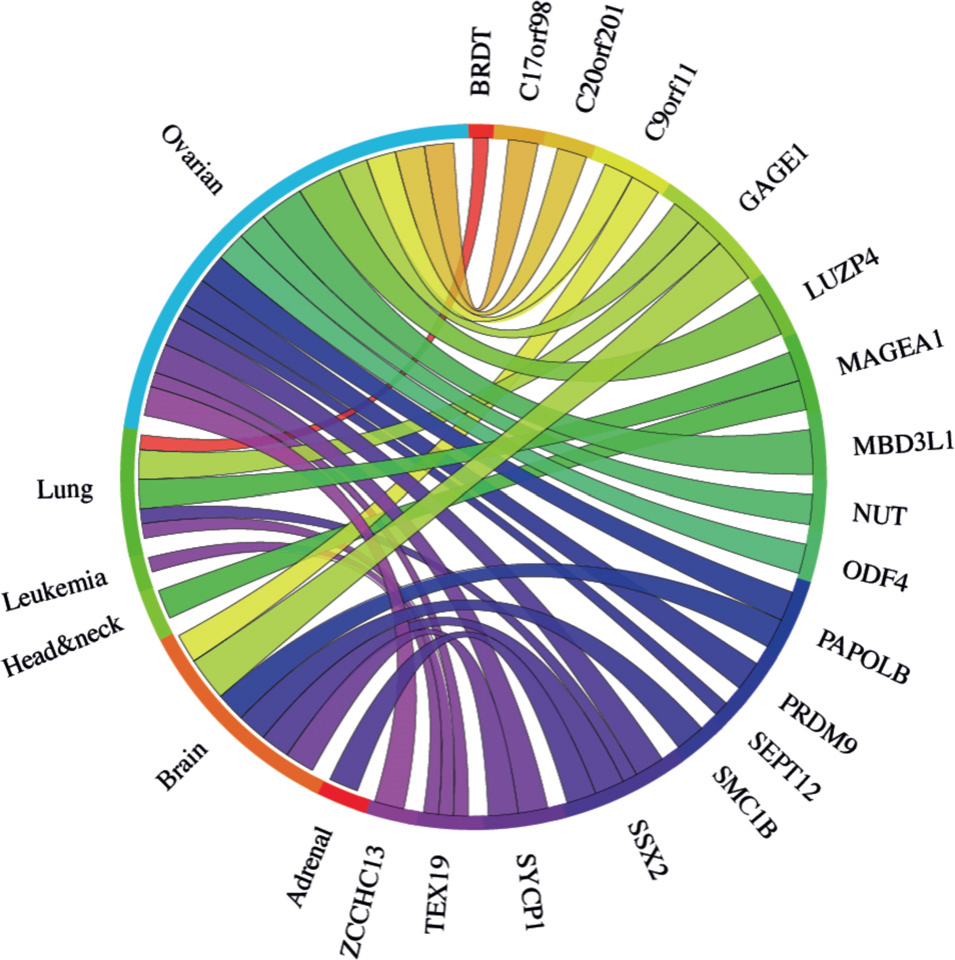
The Circos plot showing the meta-change in gene expression in relation to corresponding cancer types (ascribed by tissue type) for the 25 meiCT genes and the 3 known X-CT genes (*MAGE-A1, GAGE1, SSX2*) covered by array sets. 15 of the represented meiCT genes exhibit an up regulation in combined data set meta-analyses. Each connection between a gene and a cancer type indicates a statistically significant mean up regulation for that cancer type derived from a number of combined array studies for cancer tissue *vs*. normal tissue. The weight of the connection corresponds to the magnitude of the meta-change in gene expression.

To explore the clinical relevance of the 33 meiCT genes we have identified, we developed a meta-analysis pipeline for patient-derived cancer microarray data including 13 cancer types (Fig. 2; support text to Fig. 2 is given in Supplementary Information; Supplementary Table S3). We analysed the meta-change in gene expression of patient-derived, untreated cancerous tissues compared to normal tissues for the candidates as well as for 3 known X-CT control genes (*MAGE-A1, GAGE, SSX2*) in a total of 80 microarray data sets (Supplementary Table S3). Of the 33 candidates, 25 were covered by the array sets and could be evaluated (Supplementary Table S4). This revealed that 15 of the meiCT genes exhibited statistically significant, cancer-specific mean up regulation in at least one cancer type for combined data sets for specific cancer types where enough clinically-derived data sets were available (Fig. 4; Supplementary Table S4). The Circos plot (Fig. 4) shows the meta-change in gene expression in relation to the corresponding cancer type. This provides evidence that the meiCT genes are expressed in clinically-relevant material and shows examples of more extensive tumour expression patterns. Some notable patterns emerge from these analyses; firstly, many of the meiCT genes show a mean up regulation in ovarian, brain and lung cancers; secondly, a number of cancer types exhibit no mean up regulation of any of the analysed meiCT genes, these include breast and colorectal, for which 11 and 13 microarray data sets were available. However, a limited number of microarray data sets were available for many cancer types and thus designating cancer specificity from these data has limitations.

**Figure 5:**
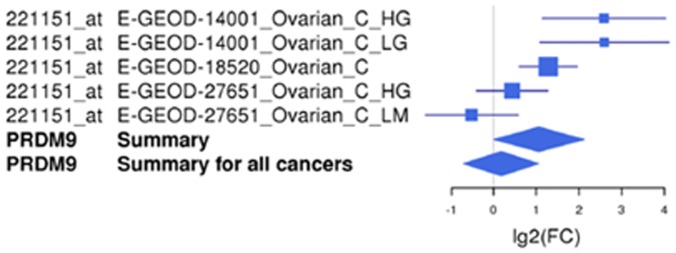
An example of a Forest plot for a meiCT gene, *PRDM9*. *PRDM9* is up regulated in one cancer type, ovarian cancer, according to the microarray meta-analysis. The Forest plot shows the log 2-fold change values for the individual studies as well as the total values for ovarian cancer and for all cancer types combined. Each study is illustrated by a square; the position on the x-axis representing the measure estimate (lg2FC ratio), the size proportional to the weight of the study, and the horizontal line through it reflecting the confidence interval of the estimate.

Whilst a significant mean up regulation is observed for a number of genes in combined data sets for distinct cancer types (Fig. 4), this does not reflect a uniform up regulation of a specific gene in all samples for a given cancer type. For example, *PRDM9* exhibits a significant mean up regulation in the ovarian cancer microarray sets used (Fig. 4); however, it is not significantly up regulated in all the individual cancer samples tested, despite the significant mean elevation (Fig. 5). This indicates that these markers may not be universally up regulated in specific cancer sub-types or cancer samples. Extending this, we determined that a further number of clinically-derived cancer samples exhibited up regulation of a wider range of the 25 meiCT genes represented on the arrays in individual (not combined) cancer data sets (cancer *vs*. normal) (Supplementary Fig. S2A) indicating the meiCT genes are expressed in clinically-relevant samples covering a broad range of cancer types.

**Figure 6:**
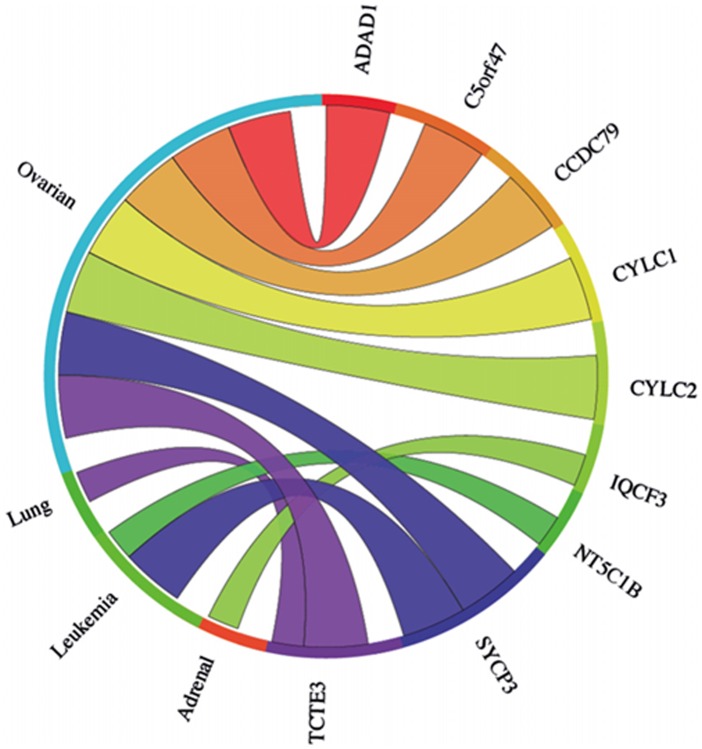
The Circos plot showing the meta-change in gene expression in relation to corresponding cancer types for the 21 genes which gave a testis-only expression profile following RT-PCR analysis and are represented on microarrays (Supplementary Table S2). 9 of the 21 genes show significant up regulation for combined cancer data sets. Each connection between a gene and a cancer type indicates a statistically significant mean up regulation for that cancer type derived from a number of combined array studies for cancer tissue *vs*. normal tissue. The weight of the connection corresponds to the magnitude of the meta-change in gene expression.

The meta-analysis approach was extended to address whether any of the 29 genes ascribed as testis-specific (no evidence for expression in any of the cancer cells we tested) by RT-PCR analysis, were up regulated in the clinically-derived microarray data sets. Of the 29 genes, 21 were represented on the arrays (Supplementary Table S2). Meta-analysis of combined cancer data sets revealed that 9 of these genes showed a significant mean up regulation in leukaemias and lung and ovarian cancers (Fig. 6). These findings indicate that these further 9 genes qualify as meiCT genes, bringing the total number of meiCT genes identified in this study to 42 (Supplementary Table S5), many of which are novel genes which have not been classified as cancer biomarkers. Additionally, analysis of individual (not combined) cancer data sets revealed up regulation of 19 of the 21 genes in a broader range of cancer types (Supplementary Fig. S2B), indicating that a further 10 genes could be considered as meiCT genes.

## DISCUSSION

The restricted regulation of CT genes has resulted in the emergence of their associated antigens as important oncological biomarkers. However, the classification of CT genes remains fraught with difficulties and it has been proposed that a uniform classification is premature until a greater insight into the biology and clinical importance of CT genes is revealed [25]. Here we add a new group of genes to this expanding family, the meiCT genes, which not only have expression restricted to the testis, but are likely to be further restricted to the highly immunologically privileged meiotic spermatocytes. These are more widely represented on the autosomes than previously characterised CT genes (45 of the 52 meiCT genes are autosomally encoded; Supplementary Table S5) and their identification opens up new possibilities in terms of both tumour distribution and oncogenic activities. Analysis of the meiCT genes demonstrates that these are expressed in a wide range of cancer types. For example, our RT-PCR validation demonstrates expression of a number of meiCT genes, including *PRDM9*, in lymphoma and leukaemia lines (Fig. 1A; Fig. 3). Of the 46 genes subjected to meta-analysis (combined cancer data sets), 20 were expressed in ovarian cancers. The use of CT antigens for immunotherapies to treat ovarian cancers has yielded positive results (for example, see 44), and so the finding that the meiCT genes are extensively expressed in ovarian cancers could provide an additional suite of markers for a tumour type which is amenable to immunotherapeutic approaches. In addition to the meta-analysis, study of individual cancer data sets (cancer *vs*. normal) suggested expression of additional meiCT genes (42 out of the 44 represented on microarrays) in a broader range of cancer sub-types (Fig. 6; Supplementary Fig. S2; Supplementary Table S5); however, the extent to which these single data set analyses reflect extensive expression in a given cancer type will rely on the generation of further clinically-derived data sets and their subsequent analyses, whereas meta-analyses are generally accepted to indicate a more precise and reliable estimate of gene expression for a given cancer type.

In our original computational analysis we applied relatively stringent conditions to the classification of the meiCT genes. We only selected candidates for validation which were not represented in EST libraries of any non-testis / non-CNS normal tissue types. Chen and co-workers [26] challenged EST data sets with testis-specific genes identified by massive parallel sequencing and retained those genes with expression in one or two other non-testis normal tissues. Indeed, when we re-ran our analysis setting the criteria with this same lower stringency we identified a significant number of additional candidates. We took the more stringent approach so as to target meiCT genes which were tightly restricted CT genes. However, on validation we revealed a number of meiCT genes which fall into the CT (and CT/CNS) selective class. This might be due to the nature of the so-called normal tissue; as in our case, RNA from many normal tissues are extracted from tissue obtained *post mortem* and are often pooled from tissues from a number of individuals, many of whom were aged at time of death. It remains a possibility that some of these tissues had undergone undiagnosed neoplastic change and might have been aberrantly expressing one or more of the candidate genes. In support of this, Chen and co-workers [26] observed expression of some genes in tissues from one panel of normal tissues, but detected no measurable expression in similar tissue types from a distinct second source. Thus, genes which exhibit meiCT selective profiles, such as *TEX19*, might indeed be CT restricted genes and be of clinical use.

### Meiotic chromosome regulators as CT genes

Here we have identified a number of previously uncharacterised genes as meiCT genes; for example, *C12orf12*. However, we also find that a number of relatively well characterised meiotic genes are meiCT genes. It has been previously proposed that the aberrant expression of CT genes may have an oncogenic effect [18,32] and indeed, aberrant expression of germ line genes in *Drosophila* contributes to malignant growth [45]; when this idea is applied to the genes identified here it opens up some interesting possibilities, which might indicate that the meiCT genes might not only be oncogenic, but might also provide drug targeting opportunities. For example, the meiotic cohesin genes *RAD21L* and *SMC1beta* may produce proteins which are incorporated into functional cohesin complexes within mitotically dividing tissues; this may not only result in aberrant modulation of chromosome segregation resulting in genome instability, but might also provide a cancer cell-specific drug target to inhibit chromosome segregation.

The expression of the meiotic recombination hotspot activator gene *PRDM9* is intriguing as the gene product is a sequence-specific zinc finger histone methyltransferase known to regulate the epigenetic programme for hotspot chromatin activation [46,47] and in mice the orthologue, Meisetz, has a function in transcriptional regulation where it activates expression of the testis-specific *RIK* gene, amongst others [48]. We could find no evidence that any of the human orthologues of *RIK* were differentially activated in cancer cells expressing *PRDM9*, but the possibility remains that active PRDM9 protein in somatic cells might trigger unscheduled transcriptional activity and/or generate regions with altered chromatin structure which could form unstable chromatin lesions, both of which could be oncogenic in nature.

In addition to identifying the meiCT genes, we found expression in non-testis tissues of a number of genes which are reported as meiosis-specific, including *REC8* and *STAG3*. This is not inconsistent with previous studies, where these genes are reported to be up regulated in the testis and are not testis-restricted. Why might some genes, which encode meiosis-specific functions, be less tightly regulated than others? The answer to this could come from studies in the fission yeast were the production of Rec8 protein in mitotic cells is inhibited by specific post-transcriptional mRNA degradation [49,50]. If an analogous system were operating in mammals then many CT antigen genes might be missed using transcriptional profiling alone; *REC8* and *STAG3* might prove to be good genes on which to test this idea. This raises the possibility that CT antigens can be generated not only by transcriptional dysfunction, but also by the de-regulation of translational repression programmes which ensure spermatocyte-/testis-specific translation.

### Conclusions

Here we have characterised a sub-class of a clinically-important family of genes and identified a large number of previously unclassified/uncharacterised genes as potential clinically-relevant cancer biomarkers. Their identification also exposes a new cohort of genes which might have oncogenic characteristics, whose protein products might not only serve as targets for immune therapeutics, but also as new drug targets and oncogenic drivers.

## MATERIALS AND METHODS

### Cell lines and cell culture

The NTERA-2 (clone D1) cell line was gifted by Prof. P.W. Andrews (University of Sheffield) and are regularly authenticated within the group using standard antibody tests using anti-OCT4 antibodies and retinoic acid-induced differentiation. The A2780 cell line was provided by Prof. P. Workman (Cancer Research UK Centre for Cancer Therapeutics, Surrey, UK) and was authenticated at source. The following cell lines were purchased from the European Collection of Cell Cultures (ECACC); 1321N1, COLO800, COLO857, G-361, HCT116, HT29, LoVo, MM127, SW480 and T84. H460 was purchased from the American Type Culture Collection (ATCC), and the two ovarian adenocarcinoma cell lines, PEO14 and TO14, were obtained from Cancer Research Technology Ltd. Primary cultures of proliferating human prostate smooth muscle cells were obtained from PromoCell™ (C-12574). All cultures were used within a six month period of obtaining validated lines from external sources.

1321N1, A2780, NTERA-2 (clone D1) and SW480 cell lines were cultured in Invitrogens Dubeco's modified Eagle's medium (DMEM+GLATAMAX™) supplemented with 10% foetal bovine serum (FBS). COLO800, COLO857 and H460 cell lines were cultured in Invitrogens Roswell Park Memorial Institute 1640 medium (RPMI 1640)+GLUTAMAX™ with 10% FBS. PEO14 and TO14 cell lines were cultured in RPMI 1640+GLUTAMAX™ supplemented with 10% FBS and 2 mM sodium pyruvate, and MM127 was cultured in RPMI 1640+GLUTAMAX™ supplemented with 10% FBS and 25 mM HEPES. Invitrogens McCoy's 5A medium+GLUTAMAX™ supplemented with 10% FBS was used to culture the G-361, HCT116 and HT29 cell lines. Ham's F12+DMEM (1:1)+GLUTAMAX™ (Invitrogen™) with 10% FBS was used to culture T84 cells.

All cell lines were grown in a 37°C incubator with 5% CO_2_, with the exception of the NTERA-2 (clone D1) cell line which was grown at 37°C with 10% CO_2_.

### cDNA construction

Total RNA preparations from the human and mouse normal tissue panels (Clontech™; 636643 and 636745 respectively). RNA from tumour tissues and cell lines were purchased from Clontech™ and Ambion™. Total RNA was also isolated from cells using TRIzol (Invitrogen). Confluent cells were collected in TRIzol reagent and incubated at room temperature for 5 minutes. Chloroform was added with vigorous shaking and incubated for 5 minutes at room temperature. The aqueous phase was transferred to a clean tube following centrifugation at 12,000 *g* for 15 minutes at 4°C. The RNA was precipitated out of solution using isopropanol (10 minutes at room temperature and centrifuged at 12,000 *g* for 20 minutes at 4°C). RNA preparations were re-suspended in RNase-free water containing DNase. The concentration and quality of RNA was measured using a NanoDrop (ND_1000). 1.0 μg of total RNA was reverse-transcribed into cDNA using SuperScript III First Strand synthesis kit (Invitrogen™) as per the manufacturer's instructions.

### RT-PCR

The sequences for each of the genes analysed were obtained from the National Center for Biotechnology (NCBI; http://www.ncbi.nlm.nih.gov/). Primers to each of the genes were designed to span exons where possible using Primer3 software (available from: www.genome.wi.mit.edu/cgi-bin/primer/primer3www.cgi; primer sequences are available upon request).

A volume of 2 μL diluted cDNA (containing ~150 ng/μl cDNA) was used for PCR in a 50 μL final volume. BioMix™ Red (Bioline™) was used for PCR amplification. Samples were amplified with a pre-cycling hold at 96°C for 5 minutes, followed by 40 cycles of denaturing at 96°C for 30 seconds, annealing at a temperature between 58-62°C for 30 seconds and extension at 72°C for 40 seconds followed by a final extension step at 72°C for 5 minutes. The products were separated on 1% agarose gels containing ethidium bromide.

### Western blot analysis

Whole cell protein lysates were prepared from cells using lysis buffer {50 mM Tris-HCl pH7.4, 200 mM sodium chloride, 0.5% Triton X-100, 1 mM AEBSF [4-(2-aminoethyl)-benzenesulfonyl fluoride] with complete, EDTA-free protease inhibitor cocktail (Roche)} and Laemmli buffer. The samples were boiled and an aliquot containing 60,000 cells was subjected to denaturing gel electrophoresis using a NuPAGE™ 4-12% Bis-Tris gel (Invitrogen™) and transferred to a PVDF membrane (Millipore™). Membranes were blocked for one hour using 1xPBST (0.3% Tween-20) containing 5% non-fat dry milk, followed by an overnight incubation at 4°C with rabbit polyclonal anti-PRDM9 antibody (Abcam; ab85654) at a dilution of 1:1,000, or mouse monoclonal anti-tubulin antibody (Sigma cat. no. T6074) at a dilution of 1:5,000, or goat polyclonal anti-lamin antibody (Santa Cruz cat. no. sc-6217) at a dilution of 1:1,000. Membranes were washed using 1xPBST and incubated with either goat, mouse or rabbit HRP-conjugated IgG antibody dependent upon the primary antibody. ECL detection reagents were then used for visulisation (SuperSignal West Pico Chemiluminescent Substrate; Thermo Scientific).

## Supplementary Figures and Tables


