# Supplementary material for: Meta-analysis of clinical data using human meiotic genes identifies a novel cohort of highly restricted cancer-specific marker genes
Source: Oncotarget. 2012 Aug 13;3(8):843–53. doi: 10.18632/oncotarget.580 (PMC3478461; doi:10.18632/oncotarget.580)
Supplement: Supplementary file 1 [file oncotarget-08-843-s001.pdf]

Meta-analysis of clinical data using human meiotic genes identifies a novel cohort of highly restricted cancer-specific marker genes - Feichtinger et al

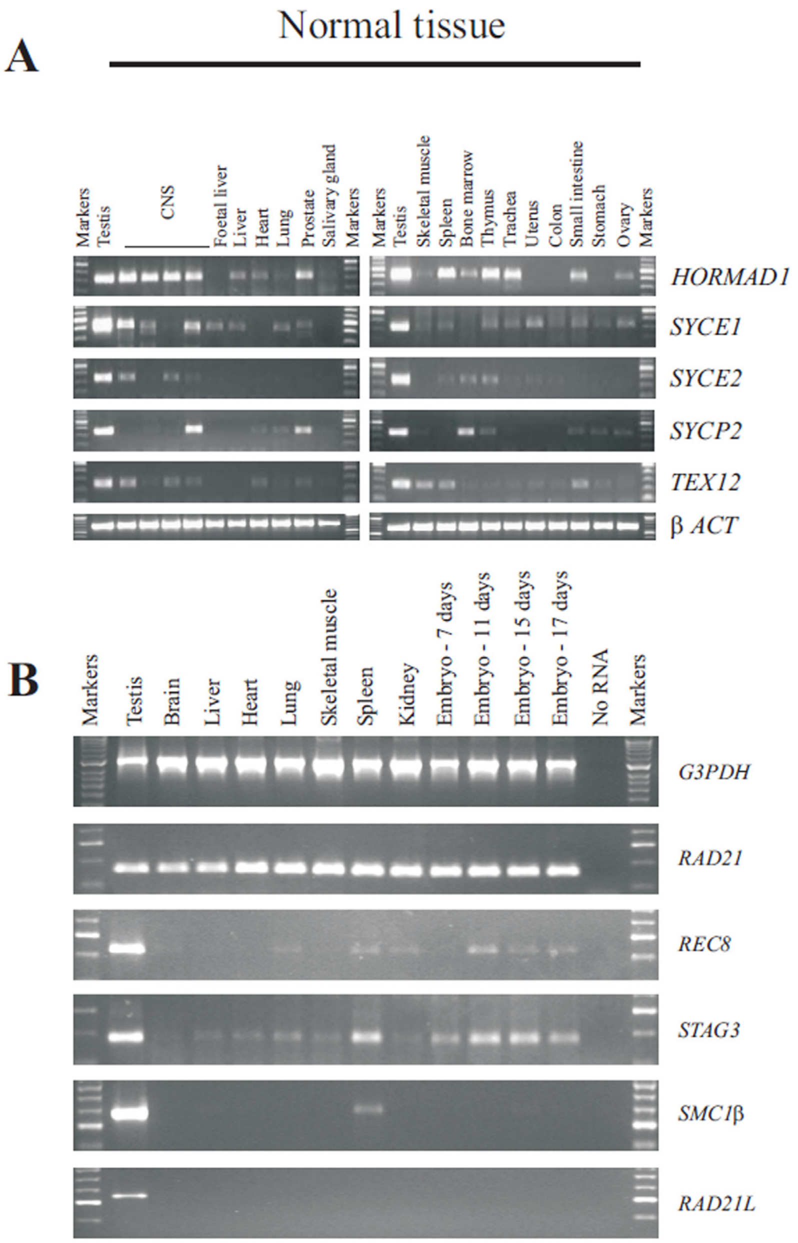

Figure S1: RT-PCR analysis of selected human and mouse meiosis-associated genes. A. Expression profiles for five human genes originally predicted to be testis-specific. The image shows agarose gels of RT-PCR products for the five genes (*HORMAD1, SYCE1, SYCE2, SYCP2, TEX12*) obtained from cDNA derived from normal human tissue RNA (obtained *post mortem*). βACT gene expression is used as a control (bottom row). A selection of bands was subjected to DNA sequencing for validation. **B.** Gene expression profiles for mouse cohesin genes. The images show agarose gels of RT-PCR products for five mouse cohesion genes (*RAD21, RAD21L, REC8, SMC1β, STAG3*). *SMC1β* and *RAD21L* show testis-selective and testis-restricted expression profiles respectively. The other three, *RAD21, REC8, STAG3*, exhibit expression in an extensive range of non-meiotic tissue types. *G3PDH* was used as a positive control for cDNA quality. A selection of bands was subjected to DNA sequencing for validation.

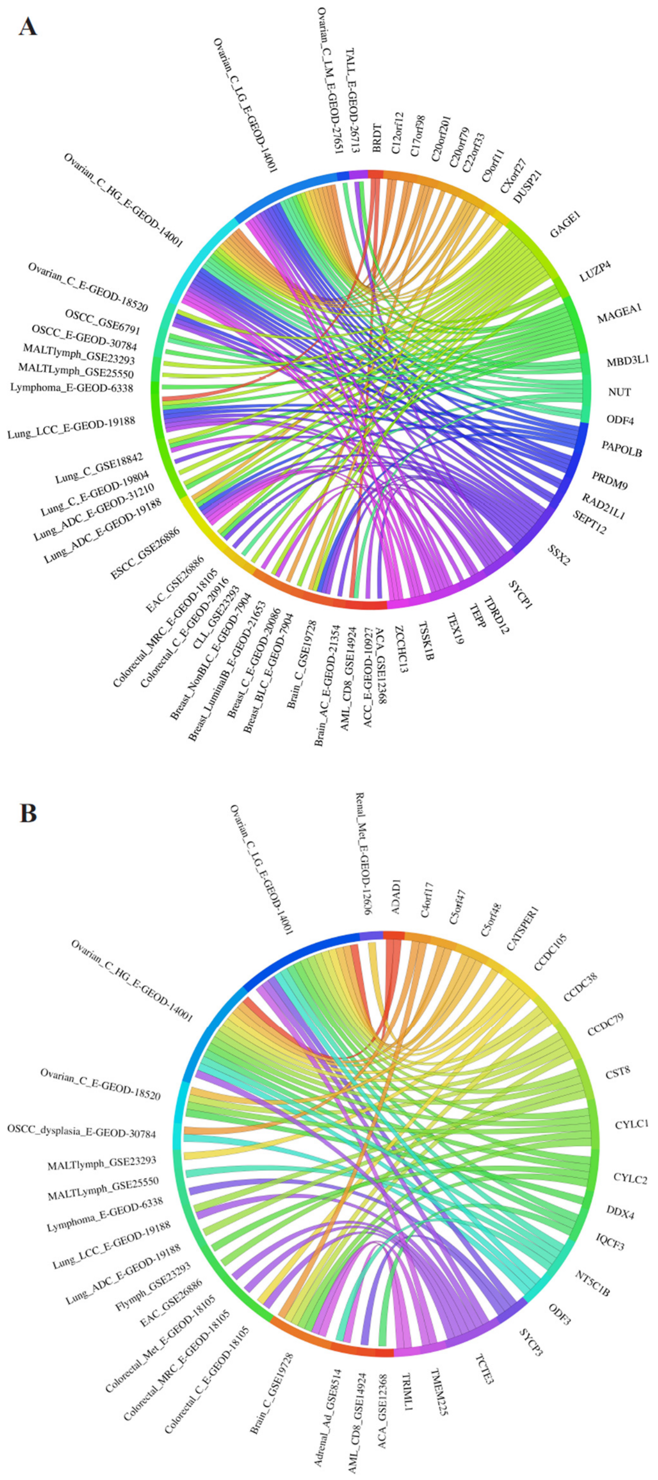

Figure S2: Circus plots for single microarray analyse. A. The Circos plot showing single microarray analysis in relation to corresponding cancer types for the 25 meiCT genes and the 3 known X-CT genes (*MAGE-A1, GAGE1, SSX2*) covered by array sets (Supporting Information Table 4). Each connection between a gene and an individual cancer type indicates a statistically significant up regulation for that cancer type derived from a single array study for cancer tissue *vs.* normal tissue. **B.** The Circos plot showing single microarray analysis for the 21 genes which gave a testis (meiosis) only gene expression profile following RT-PCR analysis and are represented on microarrays (see Supporting Information Table 2). Each connection between a gene and an individual cancer type indicates a statistically significant up regulation for that cancer type derived from a single array study for cancer tissue *vs.* normal tissue.

Table S1: List of selected meiosis-associated genes used in initial study.

Gene name	Functional role	Reference	Classification following validation
HORMAD1	Recombination partner choice regulation	Wojtasz *et al*. (2009) *PLoS Genetics* 5: e1000702<br>Shin *et al*. (2010) *PLoS Genetics* 6: e1001190	Dismissed
NUT (C15orf55)	Unknown	French (2012) *Annu Rev Patho* l 7: 247-265	Restricted CT gene
PRDM9	Meiotic hotspot regulation	Hochwagen & Marais (2010) *Curr Biol* 20: R271-274<br>Neale (2010) *Genome Biol* 11: 104	Restricted CT gene
RAD21L	Meiotic cohesin subunit	Lee & Hirano (2011) *J Cell Biol* 192:263-276<br>Ishiguro *et al*. (2011) *EMBO Rep* 12: 267-275	Restricted CT gene
REC8	Meiotic cohesin subunit	Bardhan (2010) Chromosome Res 18: 909-924	Dismissed
SMC1β	Meiotic cohesin subunit	Bardhan (2010) *Chromosome Res* 18: 909-924	Restricted CT gene
STAG3	Meiotic cohesin subunit	Bardhan (2010) Chromosome Res 18: 909-924	Dismissed
STRA8	Retinoic acid induced meiotic regulator	Anderson *et al*. (2008) *Proc Natl Acad Sci USA* 105: 14976-14980	Restricted CT gene
SYCE1	Synaptonemal complex component	Bolcun-Filas *et al*. (2009) PLoS Genetics 5: e1000393	Dismissed
SYCE2	Synaptonemal complex component	Bolcun-Filas *et al*. (2007) *J Cell Biol* 176: 741-747	Dismissed
SYCP1	Synaptonemal complex component	Pousette *et al*. (1997) *Hum Reprod* 12: 2414-2417<br>Tarsounas *et al*. (1999) *J Cell Sci* 112: 423-434	Restricted CT / CNS gene
SYCP2	Synaptonemal complex component	Schalk *et al*. (1998) *Chromosoma* 107: 540-548<br>Yang *et al*. (2006) *J Cell Biol* 173: 497-507	Dismissed
TEX12	Meiotically up regulated	Hamer *et al*. (2006) *J Cell Sci* 119: 4025-4032	Dismissed
TEX19	Meiotically up regulated	Kuntz *et al*. (2008) *Stem Cells* 26: 734-744<br>Ollinger *et al*. (2008) *PLoS Genetics* 4: e1000199	Selective CT gene
TSSK1	Meiotic serine/threonine kinase	Li *et al*. (2011) *Mol Hum Reprod* 17: 42-56	Restricted CT / CNS gene

Table S2: 29 genes designated as testis only expression as measured by RT-PCR validation including their coverage on arrays.

Gene name	Ensembl ID	Unigene cluster ID	Array coverage
ADAD1	ENSG00000164113	Hs.518957	231448_at, 240299_at
ARL13A	ENSG00000174225	Hs.147237	Not on array
ARRDC5	ENSG00000205784	Hs.574574	Not on array
C4orf17	ENSG00000138813	Hs.97501	223990_at
C4orf51	ENSG00000237136	Hs.452865	Not on array
C5orf47	ENSG00000185056	Hs.131469	1557056_at, 1557057_a_at
C5orf48	ENSG00000196900	Hs.177983	237428_at
C5orf50	ENSG00000185662	Hs.591740	Not on array
C7orf72	ENSG00000164500	Hs.99248	Not on array
CATSPER1	ENSG00000175294	Hs.189105	1552335_at
CCDC38	ENSG00000165972	Hs.210377	1553893_at
CCDC79	ENSG00000177461	Hs.376505	1557620_a_at
CCDC105	ENSG00000160994	Hs.375985	1553451_at
CST8	ENSG00000125815	Hs.121602	220627_at
CYLC1	ENSG00000183035	Hs.444230	216778_s_at, 216779_at, 216809_at
CYLC2	ENSG00000155833	Hs.3232	207780_at
DDX4	ENSG00000152670	Hs.223581	221630_s_at
EFCAB9	ENSG00000214360	Hs.716824	Not on array
GLT6D1	ENSG00000204007	Hs.522491	Not on array
IQCF3	ENSG00000229972	Hs.729443	1555235_s_at, 236871_s_at
KCNU1	ENSG00000215262	Hs.13861	237273_at,
NT5C1B	ENSG00000185013	Hs.120319	1554368_at, 222203_s_at, 243100_at
ODF3	ENSG00000177947	Hs.350949	1553051_s_at, 233795_at
SYCP3	ENSG00000139351	Hs.506504	231618_s_at,
SUNC1 (SUN3)	ENSG00000164744	Hs.406711	1553599_a_at, 241861_at
TCTE3	ENSG00000184786	Hs.733746	1554400_at, 1554401_a_at, 1557945_at, 232258_at
TMEM202	ENSG00000187806	Hs.446069	Not on array
TMEM225	ENSG00000204300	Hs.98377	244460_at
TRIML1	ENSG00000184108	Hs.348618	1557677_a_at

Table S3: List of the 80 data sets supported by the microarray meta-analysis including the corresponding cancer type, cancer sub-type and tissue type.

Data set name	Cancer type	Cancer sub-type	Tissue
E-GEOD-10927_ACA	Adrenal cancer	Adenoma	Adrenal gland
E-GEOD-10927_ACC	Adrenal cancer	Carcinoma	Adrenal gland
GSE12368_ACA	Adrenal cancer	Adenoma	Adrenal gland
GSE12368_ACC	Adrenal cancer	Carcinoma	Adrenal gland
GSE8514_Adrenal_Ad	Adrenal cancer	Adenoma	Adrenal gland
E-GEOD-21354_Brain_AC	Brain cancer	Sarcoma	Brain
E-GEOD-21354_Brain_EM	Brain cancer	Sarcoma	Brain
E-GEOD-21354_Brain_OG	Brain cancer	Sarcoma	Brain
E-MEXP-2351_Brain_AC	Brain cancer	Sarcoma	Brain
GSE19728_Brain_C	Brain cancer	Sarcoma	Brain
E-GEOD-20086_Breast_C	Breast cancer	Carcinoma	Breast
E-GEOD-21653_Breast_BLC	Breast cancer	Carcinoma	Breast
E-GEOD-21653_Breast_ERBB2	Breast cancer	Carcinoma	Breast
E-GEOD-21653_Breast_LuminalA	Breast cancer	Carcinoma	Breast
E-GEOD-21653_Breast_LuminalB	Breast cancer	Carcinoma	Breast
E-GEOD-22544_Breast_C	Breast cancer	Carcinoma	Breast
E-GEOD-5764_Breast_IDC	Breast cancer	Carcinoma	Breast
E-GEOD-5764_Breast_ILC	Breast cancer	Carcinoma	Breast
E-GEOD-7904_Breast_BLC	Breast cancer	Carcinoma	Breast
E-GEOD-7904_Breast_BRCA1	Breast cancer	Carcinoma	Breast
E-GEOD-7904_Breast_NonBLC	Breast cancer	Carcinoma	Breast
E-GEOD-18105_Colorectal_C	Colorectal cancer	Carcinoma	Colon
E-GEOD-18105_Colorectal_Met	Colorectal cancer	Metastasis	Colon
E-GEOD-18105_Colorectal_MRC	Colorectal cancer	Metastasis	Colon
E-GEOD-20916_Colorectal_Ad	Colorectal cancer	Adenoma	Colon
E-GEOD-20916_Colorectal_ADC	Colorectal cancer	Carcinoma	Colon
E-GEOD-20916_Colorectal_C	Colorectal cancer	Carcinoma	Colon
E-GEOD-20916_Colorectal_Ep_Ad	Colorectal cancer	Adenoma	Colon
E-GEOD-20916_Colorectal_Ep_C	Colorectal cancer	Carcinoma	Colon
E-GEOD-20916_Colorectal_Muc_Ad	Colorectal cancer	Adenoma	Colon
E-GEOD-20916_Colorectal_Muc_C	Colorectal cancer	Carcinoma	Colon
E-GEOD-23878_Colorectal_C	Colorectal cancer	Carcinoma	Colon
E-GEOD-4183_Colorectal_C	Colorectal cancer	Carcinoma	Colon
E-GEOD-4183_Colorectal_PreAd	Colorectal cancer	Adenoma	Colon
E-GEOD-12452_NPC	Head and neck cancer	Carcinoma	Nasopharynx
E-GEOD-17351_ESCC	Head and neck cancer	Carcinoma	Esophagus
E-GEOD-30784_OSCC	Head and neck cancer	Carcinoma	Oral tissue
E-GEOD-30784_OSCC_dysplasia	Head and neck cancer	Carcinoma	Oral tissue
GSE6791_OSCC	Head and neck cancer	Carcinoma	Oral tissue
GSE26886_EAC.txt	Head and neck cancer	Carcinoma	Esophagus
GSE26886_ESCC.txt	Head and neck cancer	Carcinoma	Esophagus
E-GEOD-24739_CML	Leukemia	Hematological malignancy	Blood/bone marrow
E-GEOD-26713_TALL	Leukemia	Hematological malignancy	Blood/bone marrow
GSE14924_AML_CD4	Leukemia	Hematological malignancy	Blood/bone marrow
GSE14924_AML_CD8	Leukemia	Hematological malignancy	Blood/bone marrow
E-GEOD-19188_Lung_ADC	Lung cancer	Carcinoma	Lung
E-GEOD-19188_Lung_LCC	Lung cancer	Carcinoma	Lung
E-GEOD-19188_Lung_SCC	Lung cancer	Carcinoma	Lung
E-GEOD-19804_Lung_C	Lung cancer	Carcinoma	Lung
E-GEOD-31210_Lung_ADC	Lung cancer	Carcinoma	Lung
GSE18842_Lung_C	Lung cancer	Carcinoma	Lung
E-GEOD-35331_Flymph	Lymphoma	Hematological malignancy	Blood/bone marrow
E-GEOD-6338_Lymphoma	Lymphoma	Hematological malignancy	Lymph node
E-MEXP-2957_CLL	Lymphoma	Hematological malignancy	Blood/bone marrow
GSE23293_CLL	Lymphoma	Hematological malignancy	Blood/bone marrow
GSE23293_Flymph	Lymphoma	Hematological malignancy	Blood/bone marrow
GSE23293_MALTLymph	Lymphoma	Hematological malignancy	Blood/bone marrow
GSE25550_MALTLymph	Lymphoma	Hematological malignancy	Spleen
GSE26725_CLL	Lymphoma	Hematological malignancy	Blood/bone marrow
E-GEOD-14001_Ovarian_C_HG	Ovarian cancer	Carcinoma	Ovary
E-GEOD-14001_Ovarian_C_LG	Ovarian cancer	Carcinoma	Ovary
E-GEOD-18520_Ovarian_C	Ovarian cancer	Carcinoma	Ovary
E-GEOD-27651_Ovarian_C_HG	Ovarian cancer	Carcinoma	Ovary
E-GEOD-27651_Ovarian_C_LM	Ovarian cancer	Carcinoma	Ovary
E-GEOD-22780_Pancreatic_ADC	Pancreatic cancer	Carcinoma	Pancreas

Table S3. List of the 80 data sets supported by the microarray meta-analysis including the corresponding cancer type, cancer sub-type and tissue type. (Continued)

Data set name	Cancer type	Cancer sub-type	Tissue
GSE15471_Pancreatic_C	Pancreatic cancer	Carcinoma	Pancreas
E-GEOD-17906_Prostate_C	Prostate cancer	Carcinoma	Prostate
E-GEOD-30522_Prostate_C	Prostate cancer	Carcinoma	Prostate
E-GEOD-12606_Renal_C	Renal cancer	Carcinoma	Kidney
E-GEOD-12606_Renal_Met	Renal cancer	Metastasis	Kidney
E-TABM-282_Renal_C	Renal cancer	Carcinoma	Kidney
GSE11151_CRCC	Renal cancer	Carcinoma	Kidney
GSE11151_PRCC	Renal cancer	Carcinoma	Kidney
GSE11151_Renal_C	Renal cancer	Carcinoma	Kidney
GSE11151_Renal_Onc	Renal cancer	Carcinoma	Kidney
E-GEOD-6004_Thyroid_C_Center	Thyroid cancer	Carcinoma	Thyroid gland
E-GEOD-6004_Thyroid_C_Invasive	Thyroid cancer	Carcinoma	Thyroid gland
E-MEXP-2442_Thyroid_ATC	Thyroid cancer	Carcinoma	Thyroid gland
E-MEXP-2442_Thyroid_FAd	Thyroid cancer	Adenoma	Thyroid gland
E-MEXP-2442_Thyroid_FCarc	Thyroid cancer	Carcinoma	Thyroid gland

Abbreviation	Meaning
AC	Astrocytoma
ACA	Adrenocortical adenoma
ACC	Adrenocortical carcinoma
Ad	Adenoma
ADC	Adenocarcinoma
AdvHCC	Advanced hepatocellular carcinoma
ATC	Thyroid anaplastic carcinoma
BLC	Basal-like cancer
BM	Bone marrow
BRCA1	BRCA1-associated
C	Cancer
Center	Center area
CLL	Chronic lymphocytic leukemia
CML	Chronic myelogenous leukemia
CRCC	Chromophobe renal cell cancer
EAC	Esophageal adenocarcinoma
EarlyHCC	Early hepatocellular carcinoma
ED	Ependymoma
Ep	Epithelium
ESCC	Esophageal squamous cell carcinoma
FAd	Follicular adenoma
FCarc	Follicular carcinoma
Flymph	Follicular lymphoma
HG	High grade
IDC	Invasive ductal carcinoma
ILC	Invasive lobular carcinoma
Invasive	Invasive area
LCC	Large-cell carcinoma
LG	Low grade
LM	Low-malignant
Met	Metastatic
MRC	Metastatic recurrence
Muc	Mucosa
Neo	Neoplasm
NPC	Nasopharyngeal carcinoma
OG	Oligodendro-glioma
Onc	Oncocytoma
OSCC	Oral squamous cell carcinoma
OTSCC	Oral tongue squamous cell carcinoma
PRCC	Papillary renal cell cancer
PreAd	Precancerous adenoma
PTC	Papillary thyroid cancer
SCC	Squamous cell carcinoma
TALL	T-cell acute lymphoblastic leukemia
VAdvHCC	Very advanced hepatocellular carcinoma
VEarlyHCC	Very early hepatocellular carcinoma

Table S4: List the 33 candidates and three control CTA genes (*MAGE-A1, GAGE, SSX2*) including their coverage on the arrays and their cancer-specific up regulation according to the microarray meta-analysis. For each candidate the meta-log 2-fold change (log2FC) and the confidence intervals (CI left, CI right) are stated.

Gene	Ensembl ID	Array Coverage	Cancer type	log2FC	CI left	CI right
ACTRT1	ENSG00000123165	Not on array				
BRDT	ENSG00000137948	206787_at	Lung cancer	0.97	0.22	1.73
C12orf12	ENSG00000197651	236968_at				
C15orf55	ENSG00000184507	1564603_at, 231338_at	Ovarian cancer	1.40	0.33	2.47
C17orf98	ENSG00000214556	244316_at	Ovarian cancer	1.14	−0.17	2.44
C19orf67	ENSG00000188032	Not on array				
C1orf65	ENSG00000178395	1552391_at				
C20orf201	ENSG00000171695	1554977_at	Ovarian cancer	1.05	0.11	1.99
C20orf79	ENSG00000132631	231134_at				
C22orf33	ENSG00000185264	231617_at				
C9orf11	ENSG00000120160	1554981_at, 1554982_a_at,	Ovarian cancer	1.80	0.56	3.05
		232868_at	Brain cancer	1.83	−0.30	3.95
CXorf27	ENSG00000187516	215142_at				
DUSP21	ENSG00000189037	220515_at				
FABP9	ENSG00000205186	Not on array				
FTMT	ENSG00000181867	Not on array				
GAGE1	ENSG00000205777	207086_x_at, 207739_s_at,	Ovarian cancer	1.23	−0.30	2.76
		208155_x_at, 208283_at	Lung cancer	1.13	0.30	1.95
			Brain cancer	2.38	0.30	4.47
IL31	ENSG00000204671	Not on array				
LUZP4	ENSG00000102021	220665_at	Ovarian cancer	2.73	1.38	4.08
MAGEA1	ENSG00000198681	207325_x_at	Head and neck cancer	1.21	0.15	2.28
			Lung cancer	1.48	0.67	2.30
MAGEB5	ENSG00000188408	Not on array				
MBD3L1	ENSG00000170948	1552458_at, 1552459_a_at	Ovarian cancer	2.56	1.45	3.66
ODF4	ENSG00000184650	1552408_at, 1552409_a_at	Ovarian cancer	1.09	−0.06	2.23
PAPOLB	ENSG00000218823	208271_at, 242158_at	Ovarian cancer	1.70	0.64	2.76
			Brain cancer	1.36	0.32	2.40
PFN3	ENSG00000196570	Not on array				
PRDM9	ENSG00000164256	221151_at	Ovarian cancer	1.06	0.01	2.12
RAD21L1	ENSG00000244588	215917_at, 234662_at				
SEPT12	ENSG00000140623	230947_at	Ovarian cancer	0.75	0.04	1.45
SMC1β	ENSG00000077935	1553249_at	Brain cancer	1.39	−0.46	3.24
SSX2	ENSG00000241476	207493_x_at, 210497_x_at,	Adrenal cancer	1.09	0.08	2.10
		215881_x_at, 216471_x_at	Ovarian cancer	1.07	−0.19	2.34
			Lung cancer	0.89	0.28	1.51
			Brain cancer	1.50	0.33	2.67
STRA8	ENSG00000146857	Not on array				
SYCP1	ENSG00000198765	206740_x_at, 216917_s_at	Ovarian cancer	1.64	0.51	2.77
			Brain cancer	1.78	0.12	3.45
TDRD12	ENSG00000173809	215356_at				
TEPP	ENSG00000159648	240119_at				
TEX19	ENSG00000182459	241367_at	Ovarian cancer	0.96	0.14	1.78
			Lung cancer	0.47	0.01	0.93
			Leukemia	0.47	0.01	0.93
TSSK1B	ENSG00000212122	211694_at				
ZCCHC13	ENSG00000187969	1554210_at	Ovarian cancer	1.41	0.40	2.42

Table S5: Full list of all 52 meiCT genes indicating the method used to designate them and their classification from the EST screening of the original 375 human orthologues of the mouse meiosis-specific genes.

Gene name	Chromosome	Original EST class	Method of meiCT designation	CT class
ACTRT1	X	1	R, M	CT restricted
ADAD1	4	3	M	CT restricted*
BRDT	1	3	R, M	CT restricted
C1orf65	1	3	R	CT restricted
C4orf17	4	2	S	CT restricted*
C5orf47	5	2	M	CT restricted*
C5orf48	5	2	S	CT restricted*
C9orf11	9	2	R, M	CT/CNS selective
C12orf12	12	2	R, S	CT restricted
C17orf98	17	1	R, M	CT restricted
C19orf67	19	2	R	CT restricted
C20orf79	20	2	R, S	CT restricted
C20orf201	20	2	R, M	CT/CNS restricted
C22orf33	22	2	R, S	CT restricted
CXorf27	X	2	R, S	CT/CNS selective
CATSPER1	11	3	S	CT restricted*
CCDC38	12	2	S	CT restricted*
CCDC79	16	2	M	CT restricted*
CCDC105	19	2	S	CT restricted*
CST8	20	2	S	CT restricted*
CYCL1	X	2	M	CT restricted*
CYCL2	9	2	M	CT restricted*
DDX4	5	2	S	CT restricted*
DUSP21	X	2	R, S	CT selective
FABP9	4	2	R	CT selective
FTMT	5	2	R	CT selective
IL31	12	2	R	CT selective
IQCF3	3	2	M	CT restricted*
LUZP4	X	2	R, M	CT restricted
MAGE-B5	X	2	R	CT restricted
MBD3L1	19	2	R, M	CT selective
NT5C1B	2	2	M	CT restricted*
NUT	15	Manual selection	R, M	CT restricted
ODF3	11	2	S	CT restricted*
ODF4	17	1	R, M	CT restricted
PAPOLB	7	2	R, M	CT/CNS restricted
PFN3	5	2	R	CT/CNS selective
PRDM9	5	Manual selection	R, M	CT restricted
RAD21L	20	Manual selection	R, S	CT restricted
SEPT12	16	3	R, M	CT/CNS restricted
SMC1β	22	Manual selection	R, M	CT restricted
STRA8	7	Manual selection	R	CT restricted
SYCP1	1	Manual selection	R, M	CT/CNS restricted
SYCP3	12	2	M	CT restricted*
TCTE3	6	2	M	CT restricted*
TDRD12	19	2	R, S	CT restricted
TEPP	16	2	R, S	CT selective
TEX19	17	3	R, M	CT selective
TMEM225	11	2	S	CT restricted*
TRIML1	4	3	S	CT restricted*
TSSK1B	5	Manual selection	R, S	CT/CNS restricted
ZCCHC13	X	2	R, M	CT/CNS selective

* Those genes predicted to be meiCT genes based on microarray analyses (meta or individual) have been validated for tight testis specificity by RT-PCR (see Supplementary Information Table S2).

R – Determined to be expressed in cancer samples by RT-PCR.

M – Determined to be expressed in cancer samples by microarray meta-analyses of combined microarray data sets.

S – Determined to be expressed in cancer samples by analysis of at least one individual microarray data set; these designations have the limitations imposed by statistical rigour being derived from a single microarray data set.

SUPPLEMENTARY INFORMATION: COMPUTATIONAL ANALYSES

Generation of a meiosis-specific data set

The meiosis-specific gene set was generated using: Perl 5.8.8 (available from: http://www.perl.org); and the Biomart portals (1) for GermOnline (2), MGI (3), and Ensembl (4). The initial meiosis gene set was derived from a microarray study by Chalmel *et al.* (5,6) whereby the meiotic transcriptome of mice was studied by analysing 17 somatic non-testicular control tissues, total testis, isolated seminiferous tubules as well as enriched populations of Sertoli cells, spermatogonia, pachytene spermatocytes, and round spermatids. 744 mouse genes were selected, which were found to be differentially expressed in testis, assigned to the meiotic or post-meiotic cluster as defined by Chalmel *et al.* (5,6), and not expressed in any other tissue tested. 408 human orthologues of the 744 meiosis-specific mouse genes were identified. To improve the data quality, the gene set was cross-validated with a set of human genes known to be involved in mitosis (Mitocheck) (7). The resulting 375 genes were assigned to Unigene cluster IDs.

EST analysis pipeline

The pipeline was implemented using: MySQL 5.0.77 (available from: http://www.mysql.com); and Perl 5.8.8 (available from: http://www.perl.org). The EST data derived from the Unigene database (8). For each of the 375 genes the Unigene EST profile was evaluated to determine the expression in normal and cancerous tissues. ESTs originating from cell line or uncharacterized/ mixed tissue libraries were excluded as well as ESTs showing less than 90% similarity to the corresponding human protein. Genes were sorted into 5 classes according to their expression profile: (i) testis-restricted expression in normal individuals as well as cancer expression (class 1); (ii) testis-restricted expression in normal individuals (class 2); (iii) testis and brain-restricted expression in normal individuals as well as cancer expression (class 3); (iv) testis and brain-restricted expression in normal individuals (class 4); and (v) somatic expression in normal individuals (class 5). Class 5 genes were discarded.

Microarray meta-analysis pipeline

The pipeline was implemented using: R 2.12.1 (available from: http://www.cran.r-project.org) (9); the Bioconductor package (10); MySQL 5.0.77 (available from: http://www.mysql.com); and Perl 5.8.8 (available from: http://www.perl.org).

Raw data was obtained from microarray experiments using patient-derived, untreated cancer samples with corresponding normal samples deposited in the ArrayExpress (11) or the GEO (12) repository. Data sets produced from the HG-U133 Plus 2 array from Affymetrix were selected, as this type is widely used and covers a large proportion of the human genome. Obtained data sets were sub-divided into cancer subtypes/stages as appropriate. Data sets with less than three control or cancer samples or data sets analysing tissues influenced by other diseases, fetal tissues or cancer-associated cells such as the cancer microenvironment were excluded. Only cancer types with at least two data sets could be included. This resulted in 92 data sets originating from 50 experiments and covering 13 different cancer types. The quality of the arrays was further assessed using the 'simpleaffy' package (13) and data sets with a scale factor of 3, an ActB 3':mid ratio of 3 and a GAPDH 3':mid ratio of 1.25 were selected. Based on this, 12 data sets had to be excluded completely. Individual CEL files from of 37 data sets were excluded, as they did not fulfill the quality requirements. This resulted in 80 individual cancer data sets originating from 45 experiments and covering 13 different cancer types (Supporting Information Table 3).

The raw data of all 80 data sets were re-analysed individually to assure uniformity of the analysis process. Data were pre-processed according to methods described by Hubbell *et al.* (14) using the 'affy' package (15). The data sets were filtered with the 33 candidates, the 3 control CTA genes and the 29 meiosis-specific genes respectively, in order to reduce the number of features and to enhance the statistical power. 25 of the candidates and all 3 control CTA genes were covered by the array sets (Supporting Information Table 4), and 21 of the meiosis-specific genes were covered by the array sets (Supporting Information Table 2). For computation of differentially expressed genes, the 'Limma' package (16) from Bioconductor was used, with p values adjusted for multiple testing with Benjamini and Hochberg's method to control the false discovery rate (17).

Subsequently, a meta-p value and a meta-log2-fold change value were computed for each cancer type as listed in Supplementary Table S3 using Stouffer's method (18) and weighted linear combination (19), respectively. In order to calculate a meta-p value, the individual two-sided p values were converted to one-sided p values for up and down regulation separately, as two-sided p values are oblivious to the effect direction (20). If multiple probes mapped to the same gene identifier the most extreme log 2-fold change value with its corresponding p value was selected, as it is least likely to occur by chance. We selected genes with a meta-log2-fold change > 1 or a confidence interval that does not span 0, and a meta-p value < 0.05 as potentially significant. To visualize the data of the meta-analysis, Circos plots (21) and Forest plots (22) were created.
